# Higher Serum Soluble TREM2 as a Potential Indicative Biomarker for Cognitive Impairment in Inadequately Controlled Type 2 Diabetes Without Obesity: The DOR-KyotoJ-1

**DOI:** 10.3389/fendo.2022.880148

**Published:** 2022-05-03

**Authors:** Masashi Tanaka, Hajime Yamakage, Kazuya Muranaka, Tsutomu Yamada, Rika Araki, Atsushi Ogo, Yuka Matoba, Tetsuhiro Watanabe, Miho Saito, Seiichiro Kurita, Kazuya Yonezawa, Tsuyoshi Tanaka, Masahiro Suzuki, Morio Sawamura, Morio Matsumoto, Motonobu Nishimura, Toru Kusakabe, Hiromichi Wada, Koji Hasegawa, Kazuhiko Kotani, Mitsuhiko Noda, Noriko Satoh-Asahara

**Affiliations:** ^1^ Department of Endocrinology, Metabolism, and Hypertension Research, Clinical Research Institute, National Hospital Organization Kyoto Medical Center, Kyoto, Japan; ^2^ Department of Physical Therapy, Health Science University, Yamanashi, Japan; ^3^ Department of Endocrinology and Diabetes, National Hospital Organization Nagoya Medical Center, Aichi, Japan; ^4^ Department of Diabetes and Endocrinology, National Hospital Organization National Mie Hospital, Mie, Japan; ^5^ Department of Metabolism and Endocrinology, Clinical Research Institute, National Hospital Organization Kyushu Medical Center, Fukuoka, Japan; ^6^ Department of Internal Medicine, Tokushima National Hospital, Tokushima, Japan; ^7^ Department of Internal Medicine, National Hospital Organization Kanazawa Medical Center, Ishikawa, Japan; ^8^ Department of Clinical Research, Hakodate National Hospital, Hokkaido, Japan; ^9^ Department of Endocrinology and Metabolism, National Hospital Organization Mie Chuo Medical Center, Mie, Japan; ^10^ Department of Clinical Research, National Hospital Organization Saitama Hospital, Saitama, Japan; ^11^ Department of Hematology, National Hospital Organization Shibukawa Medical Center, Gunma, Japan; ^12^ Department of Diabetes and Endocrinology, National Hospital Organization Chibahigashi National Hospital, Chiba, Japan; ^13^ Division of Translational Research, Clinical Research Institute, National Hospital Organization Kyoto Medical Center, Kyoto, Japan; ^14^ Center for Community Medicine, Jichi Medical University, Tochigi, Japan; ^15^ Department of Diabetes, Metabolism and Endocrinology, Ichikawa Hospital, International University of Health and Welfare, Chiba, Japan; ^16^ Department of Endocrinology and Diabetes, Saitama Medical University, Saitama, Japan; ^17^ Department of Metabolic Syndrome and Nutritional Science, Research Institute of Environmental Medicine, Nagoya University, Aichi, Japan

**Keywords:** glycemic control, longitudinal cohort study, serum soluble TREM2, cognitive impairment (CI), type 2 diabetes

## Abstract

**Objective:**

Type 2 diabetes is a risk factor for dementia. We investigated whether serum levels of soluble triggering receptor expressed on myeloid cell 2 (sTREM2), a soluble form of the cell surface receptor TREM2, were predictive of cognitive impairment in type 2 diabetes without obesity.

**Methods:**

A total of 166 Japanese patients with type 2 diabetes without obesity were followed-up for 2 years. We measured clinical parameters, assessed cognitive function using the mini-mental state examination (MMSE), quantified and divided serum sTREM2 levels into quartiles, and examined the longitudinal associations.

**Results:**

During the follow-up, HbA_1c_ levels were elevated in 98 patients and decreased in 68 patients. In the HbA_1c_-elevated group, higher sTREM2 levels at baseline showed a significant association with a greater tendency for reduction in MMSE scores (*P* for trend = 0.015), whereas they were not significantly associated with other examined parameters. In the HbA_1c_-decreased group, there was no significant association between sTREM2 levels at baseline and changes in MMSE scores, but higher sTREM2 levels at baseline were significantly associated with a greater tendency for reduction in waist circumference (*P* for trend = 0.027), homeostasis model assessment of insulin resistance (*P* for trend = 0.039), and sTREM2 levels (*P* for trend = 0.023).

**Conclusions:**

Glycemic control is suggested to be important in preventing cognitive impairment in patients with type 2 diabetes without obesity. Higher serum sTREM2 levels would be a predictive marker for cognitive impairment in inadequately controlled type 2 diabetes without obesity.

## Introduction

Type 2 diabetes is an epidemiological risk factor for dementia (1–3). Furthermore, the incidence of dementia (4, 5) and the prevalence of diabetes in the global aging populations are expanding worldwide (6). Therefore, there is an urgent need to improve the pathological relationship between type 2 diabetes and dementia, as well as to develop predictive markers and effective treatments for dementia in patients with type 2 diabetes.

Triggering receptor expressed on myeloid cells 2 (TREM2) is a cell surface receptor mainly expressed on myeloid cells such as monocytes, macrophages, and microglia (7, 8). TREM2 binds to various ligands (e.g., amyloid-β and apolipoprotein E) and activates the downstream signaling that modulates cellular functions, including phagocytosis and inflammation (7, 9). Although TREM2 has been closely implicated in the pathogenesis of neurodegenerative diseases, it has not been fully determined whether TREM2 exhibits a protective or detrimental role in the development and progression of the diseases (7–9).

A remarkable characteristic of TREM2 is its ability to release its ectodomain as a soluble form (sTREM2) into the extracellular space upon proteolytic cleavage (7–9). Elevation of sTREM2 in cerebrospinal fluid (CSF) has been suggested to reflect microglial activation (10–12) and associated with faster cognitive decline in individuals at the preclinical stage of Alzheimer’s disease (AD) (13). In addition, sTREM2 levels in serum were also negatively associated with cognitive function in patients with AD (14). Furthermore, our research group recently demonstrated longitudinally that higher levels of serum sTREM2 at baseline were associated with a higher risk of dementia development in a general elderly population in the Hisayama study (15). Therefore, it is suggested that serum sTREM2 as well as CSF sTREM2 would be a predictive marker for dementia.

Although type 2 diabetes is a risk factor for dementia, only a limited number of studies have ever addressed the pathophysiological significance of TREM2 in diabetes in both animal models and humans. In adipose tissue in mice, TREM2 was expressed on mature adipocyte and promoted adipogenesis which caused high-fat diet-induced obesity and insulin resistance (16), whereas another study reported a protective effect of TREM2 on insulin resistance (17). Thus, the roles of TREM2 in glucose metabolism remain controversial. Conversely, type 2 diabetes-related metabolic dysfunction altered microglial TREM2 signaling and resulted in microglial dysfunction, which in turn led to cognitive impairment (18), thereby suggesting pathological implications of TREM2 in the development of diabetes-related cognitive impairment.

In humans, TREM2 expression has also been observed in adipose tissue (19), which would suggest the potential roles of TREM2 in glucose metabolism. We recently demonstrated in the Hisayama cross-sectional study that the elevation of serum sTREM2 levels was associated with an increase in homeostasis model assessment of insulin resistance (HOMA-R) in the general population (20). Furthermore, in patients with type 2 diabetes without obesity, we found that the elevation of serum sTREM2 levels was cross-sectionally associated with cognitive impairment risk in a National Hospital Organization cohort to study obesity and diabetes (21). Since insulin resistance is implicated in cognitive impairment (22–24), these findings suggest that sTREM2 is a pathological mediator between exacerbation of glucose metabolism and cognitive impairment. Therefore, serum sTREM2 levels may be a novel marker for predicting future cognitive impairment in diabetic conditions.

Based on the evidence that type 2 diabetes is a risk factor for dementia (1–3), it is possible that the progression of cognitive impairment differs between patients with adequately and inadequately controlled type 2 diabetes. In particular in patients with type 2 diabetes without obesity, our cross-sectional study previously showed a significant association between the elevation of serum sTREM2 levels and a higher risk of cognitive impairment (21). To further address this issue, in the present study, we longitudinally investigated the association of serum sTREM2 with clinical parameters including cognitive function in patients with type 2 diabetes without obesity, in whom levels of hemoglobin A_1c_ (HbA_1c_) were elevated or decreased during the 2-year follow-up period, using a database of a cohort comprising patients with obesity and/or diabetes.

## Methods

### Study Population and Study Design

**Figure 1:**
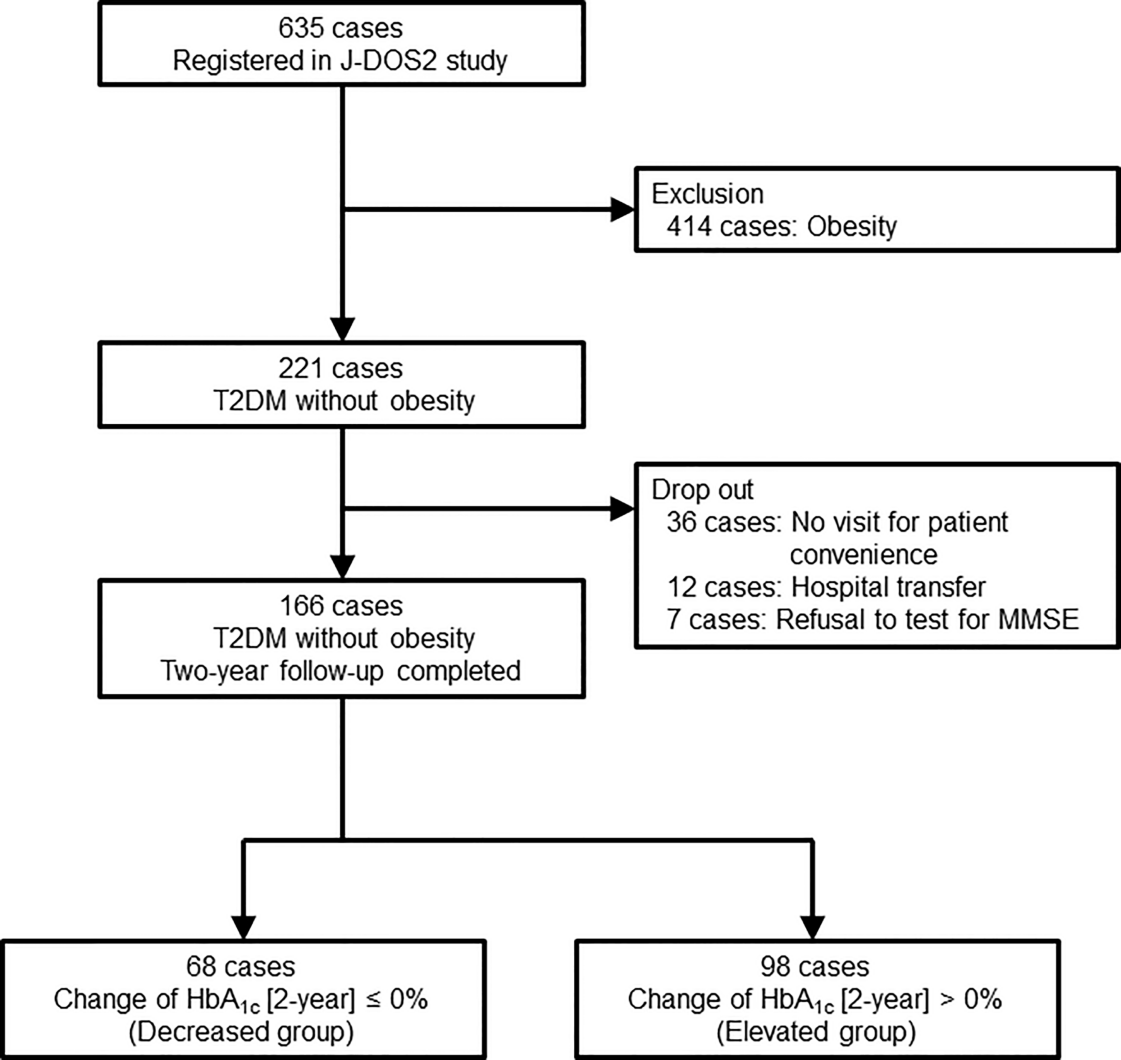
Flow chart of the study. J-DOS2, Japan Diabetes and Obesity Study2; T2DM, type 2 diabetes mellitus; MMSE, mini-mental state examination; HbA_1c_, hemoglobin A_1c_.

This study was conducted in an ongoing longitudinal multicenter prospective observational cohort study (Japan Obesity & Metabolic Syndrome Study [JOMS]/Japan Diabetes and Obesity Study2 [J-DOS2]), consisting of 11 National Hospital Organization hospitals (Kyoto, Chiba, Gunma, Aichi, Mie, Fukuoka, Ishikawa, Tokushima, Hokkaido, and Saitama), under the framework of Diabetes and Obesity Registry Kyoto in Japan (DOR-KyotoJ). A total of 635 Japanese patients with type 2 diabetes aged 20–79 years were enrolled from February 2015 to January 2017. Of these, 166 subjects without obesity underwent measurement of serum sTREM2 levels and were included in this study (**Figure 1**). Type 2 diabetes was diagnosed based on the guidelines of the Japan Diabetes Society, and obesity was defined as a BMI of ≥25 kg/m^2^, according to the Japan Society for the Study of Obesity guidelines. All the patients received diet and exercise therapies for type 2 diabetes. Candidates were not predetermined by cognition and mentation. Subjects were excluded at baseline if they had a previous history of cardiovascular diseases, other vascular diseases, severe liver dysfunction, severe renal disease (serum creatinine ≥ 265.2 μmol/L), and secondary obesity due to endocrine disorders.

This study was designed to investigate the relationship between serum sTREM2 levels and cognitive function, a primary outcome assessed by the mini-mental state examination (MMSE) longitudinally in patients with type 2 diabetes without obesity who were followed-up for 2 years. Data comprising both males and females were analyzed. Approval for the study was obtained from the Central Ethics Committee for Clinical Research at the National Hospital Organization headquarters (approval number 14-034) (21). The study was conducted in accordance with the Declaration of Helsinki and Ethical Guidelines for Medical and Health Research Involving Human Subjects. All participants provided written informed consent. This study is registered in the University Hospital Medical Information Network Clinical Trial Registry (UMIN-CTR) System (ID: UMIN000017929) (21).

### Assessment of Cognitive Function

We assessed cognitive function using MMSE-Japanese version (Nihon Bunka Kagakusha Co. Ltd., Tokyo [provided by Psychological Assessment Resources, Inc., FL, USA]), which consists of questions related to cognition (score range, 0–30; higher scores, better function) as described previously (21, 25, 26). MMSE scores of ≤26 were a cognitively impaired group (mild cognitive impairment and dementia) (21, 27), and those of ≤23 corresponded to dementia, according to the guidelines of relevant societies (the Japanese Society of Neurology, the Japanese Society of Psychiatry and Neurology, the Japan Society for Dementia Research, the Japanese Psychogeriatric Society, the Japan Geriatrics Society, and the Japanese Society of Neurological Therapeutics) (21).

### Data Collection and Measurements

We measured anthropometric and metabolic parameters in all patients at baseline and 1- and 2-year follow-up using standard procedures (21). These parameters included body weight, BMI, waist circumference (WC), systolic and diastolic blood pressures, fasting plasma glucose (FPG), HbA_1c_, serum immunoreactive insulin (IRI), HOMA-R, triglycerides, total cholesterol, high-density lipoprotein cholesterol, low-density lipoprotein cholesterol, leptin, adiponectin, and high-sensitivity C-reactive protein (hsCRP) levels.

Serum sTREM2 levels were quantified at Health Science West Japan (Kyoto, Japan) using a RayBio Human TREM-2 ELISA Kit (RayBiotech, Norcross, GA, USA), according to the manufacturer’s instructions as described previously (15, 20, 21). The patients were divided into four categories according to the serum levels of sTREM2.

### Statistical Analysis

The sample size for this study was all cases of diabetes without obesity from the database of the underlying J-DOS2 study.

The results were described as mean ± standard deviation, median [interquartile range], or the number and percentage of patients. The normality of continuous variables was checked by histograms. When the distribution was normal, the parametric method was used. However, a nonparametric method was used if the distribution was not normal. To measure the amount of change over the 2 years, we used parametric methods and determined all data to be normative.

We defined groups as “elevated” groups if the 2-year HbA_1c_ change was >0 or “decreased” groups if the 2-year HbA_1c_ change was ≤0 (decreased). In the comparison between baseline parameters in the previous two groups, we used Fisher’s exact test, unpaired t-test, or Mann–Whitney U test. Analysis of covariance with age and gender as covariates was used for intergroup comparison of the change in clinical parameters over 2 years by the increased and decreased groups of the change in HbA_1c_ or by quartiles of baseline sTREM2 values. A linear contrast test was used as a trend test. A two-sided *P*-value <0.05 was considered to show statistical significance. The statistical software was SPSS version 24.0 for Windows (IBM Japan Ltd., Tokyo, Japan).

## Results

### Baseline Characteristics

This study included 166 patients with type 2 diabetes whose HbA_1c_ levels were elevated (*n* = 98) or decreased (*n* = 68) after a 2-year follow-up, of whom 38 patients (20 [20.4%] with elevated and 18 patients [26.5%] with decreased HbA_1c_ levels) exhibited MMSE scores of ≤26. Their clinical and baseline characteristics are shown in **Table 1**. We found no significant differences in anthropometric parameters and lipid metabolism-related parameters, with the exception of triglyceride levels (lower in the HbA_1c_-elevated group than in the HbA_1c_-decreased group, *P* = 0.031) between the HbA_1c_-elevated and -decreased groups. Serum sTREM2 levels and MMSE scores did not differ significantly between the two patient groups, which was the same for glucose metabolism-related profiles except for HbA_1c_ levels (lower in the HbA_1c_-elevated group compared with those in the HbA_1c_-decreased group, *P* = 0.003). There was no significant difference in medications for type 2 diabetes (total: 84.9%) between the HbA_1c_-decreased (83.8%) and -elevated (85.7%) groups (*P* = 0.826).

**Table 1:** Baseline characteristics of patients with decreased- or elevated-HbA_1c_ after 2-year follow-up.

	Overall	HbA_1c_-decreased group	HbA_1c_-elevated group	*P*-value
N	166	68	98	
Gender (M/F)	103/63	42/26	61/37	0.999
Age (year)	70.2 ± 7.6	69.9 ± 6.7	70.3 ± 8.2	0.733
Age (n, %)				
40–49	2, 1.2	0, 0.0	2, 2.0	
50–59	12, 7.2	4, 5.9	8, 8.2	
60–69	55, 33.1	27, 39.7	28, 28.6	
70–79	97, 58.4	37, 54.4	60, 61.2	
BMI (kg/m^2^)	22.1 ± 1.9	22.3 ± 1.9	22.0 ± 2.0	0.480
WC (cm)	82.7 ± 7.7	82.2 ± 7.1	83.2 ± 8.1	0.428
SBP (mmHg)	133 ± 16	131 ± 14	134 ± 18	0.221
DBP (mmHg)	75 ± 12	74 ± 12	75 ± 12	0.379
FPG (mmol/L)	8.3 ± 2.7	8.6 ± 3.4	8.1 ± 2.0	0.182
HbA_1c_ (%)	7.2 ± 1.2	7.5 ± 1.5	6.9 ± 0.9	0.003
HbA_1c_ (mmol/mol)	55 ± 13	58 ± 16	56 ± 10	–
IRI (pmol/L)	52.9 [30.8, 79.8]	49.0 [30.1, 84.0]	56.0 [30.8, 77.0]	0.749
HOMA-R	2.6 [1.5, 4.5]	2.6 [1.4, 4.9]	2.6 [1.6, 4.3]	0.914
Triglycerides (mmol/L)	1.23 [0.86, 1.63]	1.34 [0.93, 1.85]	1.13 [0.80, 1.47]	0.031
HDL-C (mmol/L)	1.5 ± 0.4	1.4 ± 0.4	1.5 ± 0.4	0.342
LDL-C (mmol/L)	2.7 [2.3, 3.3]	2.7 [2.3, 3.3]	2.7 [2.3, 3.2]	0.499
hsCRP (mg/mL)	0.5 [0.3, 1.0]	0.5 [0.3, 1.1]	0.5 [0.3, 1.0]	0.603
sTREM2 (pg/mL)	230.8 [135.4, 425.0]	218.4 [121.1, 407.8]	244.8 [141.6, 441.4]	0.485
MMSE	29.0 [27.0, 30.0]	29.0 [26.0, 30.0]	29.0 [27.0, 30.0]	0.720
Treatment (n/%)				
Diabetes	141/84.9	57/83.8	84/85.7	0.826
Hypertension	87/52.4	37/54.4	50/51.0	0.752
Dyslipidemia	96/57.8	45/66.2	51/52.0	0.080

Data are expressed as mean ± standard deviation, median [interquartile range], or the number and percentage of patients. HbA_1c_, hemoglobin A_1c_; WC, waist circumference; SBP, systolic blood pressure; DBP, diastolic blood pressure; FPG, fasting plasma glucose; IRI, immunoreactive insulin; HOMA-R, homeostasis model assessment ratio; HDL-C, high-density lipoprotein cholesterol; LDL-C, low-density lipoprotein cholesterol; hsCRP, high-sensitive C-reactive protein; sTREM2, a soluble form of triggering receptor expressed on myeloid cells 2; MMSE, mini-mental state examination. A group with a 2-year HbA_1c_ change greater than 0 (elevated group) and with a 2-year HbA_1c_ change less than 0 (decreased) were established. P-value from Fisher’s exact test, unpaired t-test, or Mann–Whitney U test (Decreased vs. Elevated groups).

### Comparison of Changes in Parameters From Baseline to the 1-Year and 2-Year Follow-up Between HbA_1c_-Elevated and -Decreased Groups

Type 2 diabetes plays a pathological role in cognitive impairment (22–24). Therefore, we examined whether differences exist between the HbA_1c_-elevated and -decreased groups, regarding changes in parameters from baseline until 1-year and 2-year follow-up. We focused on the glucose metabolism-related profiles, levels of inflammation, serum sTREM2 levels, and MMSE scores (**Supplementary Table S1**). There was a significant difference in changes in the levels of FPG (*P* = 0.009) and HOMA-R (*P* = 0.013) between the two groups after 2 years of follow-up after adjusting for age and gender, which indicates the role of glycemic control in the HbA_1c_-decreased group compared with the HbA_1c_-elevated group. Conversely, changes in the levels of IRI (*P* = 0.467), hsCRP (*P* = 0.897), and sTREM2 (*P* = 0.975) showed no significant difference between the two patient groups. There was also no significant difference between the groups in the changes in MMSE scores (*P* = 0.099) at 2-year follow-up, although the decrease in MMSE scores had a tendency to be suppressed in the HbA_1c_-decreased group compared with the HbA_1c_-elevated group after adjusting for age and gender.

### Relationships Between sTREM2 Levels at Baseline and Changes in Parameters at 1 and 2 Years of Follow-up in HbA_1c_-Elevated and -Decreased Groups

We next investigated whether serum sTREM2 levels at baseline were associated with changes in parameters of interest from baseline to the 1-year and 2-year follow-up.

**Figure 2:**
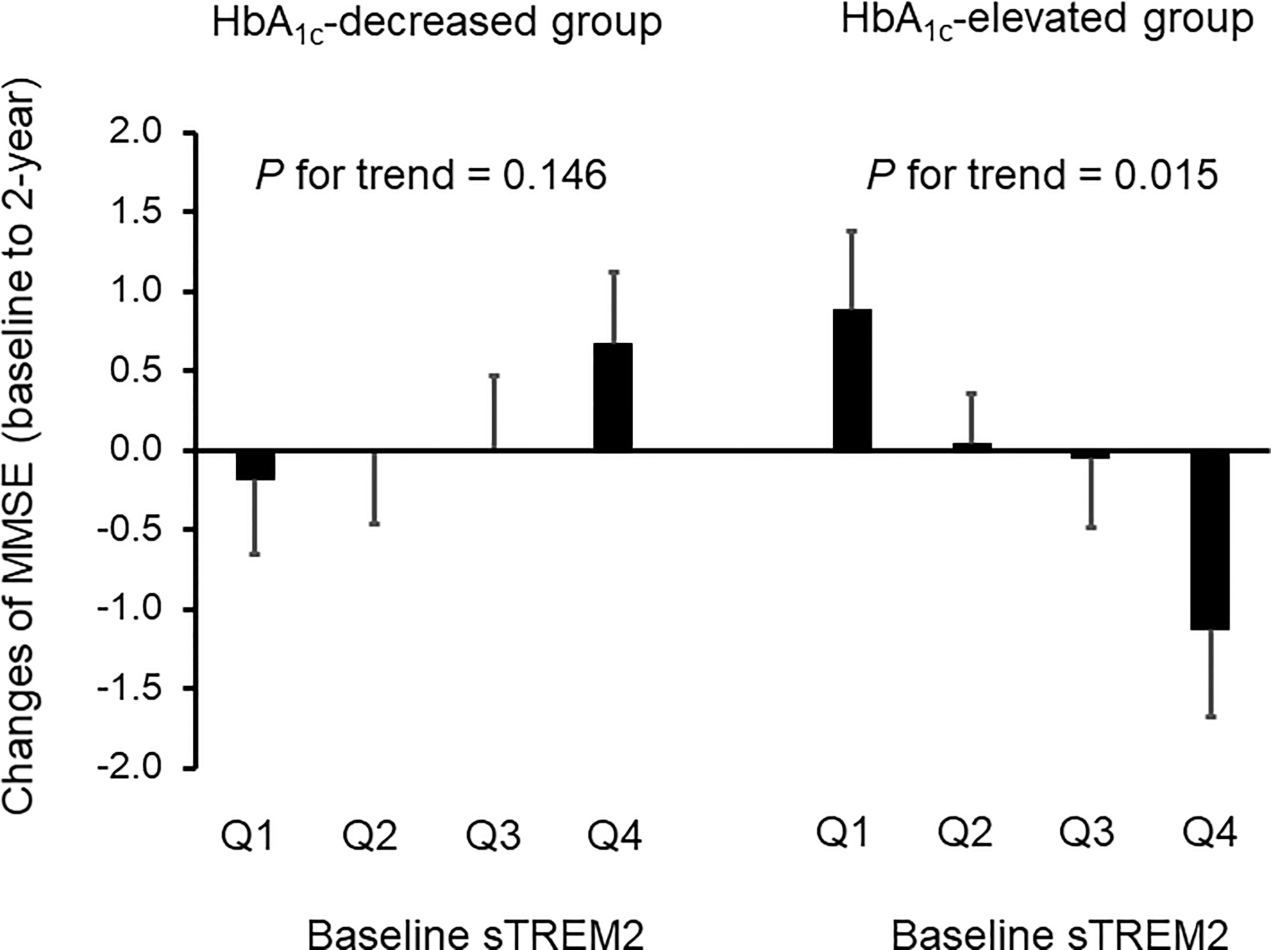
Effects of serum sTREM2 levels at baseline on cognitive impairment at 2-year follow-up in patients with adequately and inadequately controlled type 2 diabetes. The serum sTREM2 levels at baseline were divided into four categories for the HbA_1c_-decreased group (Q1, 16.2–121.0 pg/mL; Q2, 121.1–218.3 pg/mL; Q3, 218.4–407.7 pg/mL; Q4, 407.8–1223.5 pg/mL) and the HbA_1c_-elevated group (Q1, 10.7–141.5 pg/mL; Q2, 141.6–244.7 pg/mL; Q3, 244.8–441.3 pg/mL; Q4, 441.4–1728.4 pg/mL). Data are expressed as mean ± standard error. HbA_1c_, hemoglobin A_1c_; MMSE, mini-mental state examination; sTREM2, a soluble form of triggering receptor expressed on myeloid cells 2. *P*-values for trend tests were adjusted for age and gender.

In the HbA_1c_-elevated group (**Table 2**), the levels of serum sTREM2 at baseline were divided into four categories (the median value [interquartile range] in the entire group = 244.8 [141.6-441.4] pg/mL; Q1, 10.7–141.5 pg/mL; Q2, 141.6–244.7 pg/mL; Q3, 244.8–441.3 pg/mL; Q4, 441.4–1728.4 pg/mL). However, there were no significant association with changes in BMI, WC, FPG, HbA_1c_, IRI, HOMA-R, hsCRP, and sTREM2 levels after 2 years of follow-up. Conversely, higher sTREM2 levels at baseline were significantly associated with a greater tendency for reduction in MMSE scores at the 2-year follow-up after adjusting for age and gender (*P* for trend = 0.015) (**Table 2** and **Figure 2**).

**Table 2:** Relationships between sTREM2 levels at baseline and changes in parameters at 1 and 2 years of follow-up in HbA_1c_-elevated group.

	Baseline sTREM2 (pg/mL)				*P* for trend
	Q1(10.7–141.5)	Q2(141.6–244.7)	Q3(244.8–441.3)	Q4(441.4–1728.4)	
Changes from baseline to 1-year					
Δ BMI	0.2 ± 1.0	0.1 ± 0.6	−0.1 ± 1.0	−0.2 ± 2.1	0.323
Δ WC (cm)	0.2 ± 3.5	−0.3 ± 3.9	−0.3 ± 4.4	0.3 ± 3.2	0.952
Δ FPG (mmol/L)	−0.3 ± 2.7	0.7 ± 2.0	2.0 ± 2.5	1.3 ± 3.3	0.024
Δ HbA_1c_ (%)	0.1 ± 0.5	0.4 ± 0.6	0.5 ± 0.5	0.3 ± 0.7	0.179
Δ IRI (pmol/L)	6 ± 113	32 ± 77	32 ± 99	1.4 ± 19	0.896
Δ HOMA-R	0.4 ± 5.4	3.0 ± 6.9	3.2 ± 7.7	0.3 ± 0.9	0.956
Δ hsCRP (mg/mL)	−1.3 ± 4.0	0.0 ± 0.5	0.9 ± 4.9	0.3 ± 3.3	0.057
Δ sTREM2 (pg/mL)	158.4 ± 162.1	118.6 ± 148.8	158.4 ± 379.0	56.5 ± 448.4	0.461
Δ MMSE	0.3 ± 2.8	−0.1 ± 1.5	−0.3 ± 1.6	−0.5 ± 2.2	0.285
Changes from baseline to 2-year					
Δ BMI	0.5 ± 0.9	0.0 ± 0.8	0.0 ± 1.1	0.2 ± 1.6	0.591
Δ WC (cm)	1.9 ± 3.4	−0.3 ± 6.2	1.0 ± 5.6	−1.2 ± 4.4	0.291
Δ FPG (mmol/L)	0.1 ± 2.6	1.3 ± 2.5	0.6 ± 1.5	1.0 ± 3.3	0.449
Δ HbA_1c_ (%)	0.6 ± 0.6	0.9 ± 1.0	0.6 ± 0.3	0.8 ± 0.8	0.194
Δ IRI (pmol/L)	28 ± 143	−12 ± 67	39 ± 113	12 ± 38	0.892
Δ HOMA-R	2.0 ± 7.9	−0.3 ± 3.3	2.9 ± 7.0	1.4 ± 2.6	0.737
Δ hsCRP (mg/mL)	−0.9 ± 3.7	0.0 ± 0.9	1.1 ± 4.0	−0.2 ± 6.8	0.132
Δ sTREM2 (pg/mL)	238.4 ± 222.4	260.8 ± 307.1	253.0 ± 287.6	182.7 ± 568.9	0.705
Δ MMSE	0.9 ± 2.5	0.0 ± 1.5	0.0 ± 2.2	−1.1 ± 2.8	0.015

Data are expressed as mean ± standard deviation. WC, waist circumference; FPG, fasting plasma glucose; HbA_1c_, hemoglobin A_1c_; IRI, immunoreactive insulin; HOMA-R, homeostasis model assessment ratio; hsCRP, high-sensitive C-reactive protein; sTREM2, a soluble form of triggering receptor expressed on myeloid cells 2; MMSE, mini-mental state examination. Δ represents the difference between the 1- or 2-year and baseline values. P-values for trend tests were adjusted for age and gender.

In the HbA_1c_-decreased group (**Table 3**), the patients were divided into quartiles according to serum sTREM2 levels at baseline (the median value [interquartile range] in the entire group = 218.4 [121.1-407.8] pg/mL; Q1, 16.2–121.0 pg/mL; Q2, 121.1–218.3 pg/mL; Q3, 218.4–407.7 pg/mL; Q4, 407.8–1223.5 pg/mL). After adjusting for age and gender, 2-year follow-up showed no significant association between sTREM2 levels at baseline and changes in BMI, FPG, HbA_1c_, IRI, and hsCRP. Conversely, higher sTREM2 levels at baseline were significantly associated with a greater tendency for reductions in WC (*P* for trend = 0.027), HOMA-R (*P* for trend = 0.039), and sTREM2 levels (*P* for trend = 0.023) after adjusting for age and gender. On the contrary, no significant *P*-value was observed between sTREM2 levels at baseline and changes in MMSE scores at the 2-year follow-up (**Table 3** and **Figure 2**).

**Table 3:** Relationships between sTREM2 levels at baseline and changes in parameters at 1 and 2 years of follow-up in HbA_1c_-decreased group.

	Baseline sTREM2 (pg/mL)				*P* for trend
	Q1(16.2–121.0)	Q2(121.1–218.3)	Q3(218.4–407.7)	Q4(407.8–1223.5)	
Changes from baseline to 1-year					
Δ BMI	0.0 ± 1.0	−0.4 ± 1.5	0.2 ± 1.4	−0.2 ± 0.7	0.952
Δ WC (cm)	1.3 ± 3.7	1.9 ± 4.0	1.0 ± 8.9	−0.7 ± 4.1	0.310
Δ FPG (mmol/L)	−0.4 ± 3.1	0.1 ± 2.9	0.8 ± 5.2	−0.6 ± 1.5	0.941
Δ HbA_1c_ (%)	−0.2 ± 0.7	0.0 ± 0.5	0.0 ± 0.7	−0.5 ± 0.6	0.403
Δ IRI (pmol/L)	1 ± 71	40 ± 69	−26 ± 81	−4 ± 27	0.310
Δ HOMA-R	−0.9 ± 6.7	2.3 ± 5.2	−1.6 ± 6.4	0.0 ± 2.4	0.757
Δ hsCRP (mg/mL)	−0.3 ± 1.9	−0.5 ± 1.2	0.8 ± 3.1	−0.1 ± 0.8	0.434
Δ sTREM2 (pg/mL)	168.1 ± 134.4	227.0 ± 246.6	39.5 ± 238.6	−112.9 ± 308.6	<0.001
Δ MMSE	−0.3 ± 2.4	−0.1 ± 1.1	0.4 ± 2.5	0.7 ± 2.4	0.105
Changes from baseline to 2-year					
Δ BMI	0.2 ± 1.2	−0.1 ± 1.8	−0.3 ± 1.0	−0.1 ± 1.1	0.433
Δ WC (cm)	2.8 ± 5.2	2.3 ± 5.3	−1.5 ± 8.0	−1.0 ± 3.4	0.027
Δ FPG (mmol/L)	−0.4 ± 2.5	−1.4 ± 4.6	−0.1 ± 2.2	−0.7 ± 2.5	0.898
Δ HbA_1c_ (%)	−0.5 ± 0.4	−0.6 ± 0.6	−0.3 ± 0.2	−0.4 ± 0.2	0.157
Δ IRI (pmol/L)	4 ± 62	19 ± 48	−5 ± 57	−2 ± 58	0.182
Δ HOMA-R	1.2 ± 2.4	2.6 ± 5.7	−2.0 ± 5.9	−1.5 ± 2.6	0.039
Δ hsCRP (mg/mL)	−0.2 ± 2.3	−0.8 ± 1.7	0.8 ± 1.5	−0.1 ± 1.0	0.388
Δ sTREM2 (pg/mL)	280.0 ± 110.4	358.5 ± 292.3	122.8 ± 241.2	111.5 ± 313.2	0.023
Δ MMSE	−0.2 ± 1.9	0.0 ± 1.9	0.0 ± 1.9	0.7 ± 1.8	0.146

Data are expressed as mean ± standard deviation. WC, waist circumference; FPG, fasting plasma glucose; HbA_1c_, hemoglobin A_1c_; IRI, immunoreactive insulin; HOMA-R, homeostasis model assessment ratio; hsCRP, high-sensitive C-reactive protein; sTREM2, a soluble form of triggering receptor expressed on myeloid cells 2; MMSE, mini-mental state examination. Δ represents the difference between the 1 or 2-year and baseline values. P-values for trend tests were adjusted for age and gender.

## Discussion

This is the first study to show that higher levels of serum sTREM2 at baseline are significantly associated with a greater tendency for reduction in MMSE scores after 2 years of follow-up in patients with type 2 diabetes without obesity in whom HbA_1c_ levels were elevated during the 2-year follow-up period. Conversely, sTREM2 levels showed no significant association with changes in MMSE scores in patients in the HbA_1c_-decreased group. Accordingly, these results suggest that hyperglycemia and high levels of sTREM2 are closely implicated in cognitive impairment in patients with type 2 diabetes without obesity. These findings could further highlight the significance of sTREM2, as well as hyperglycemia, as an effective target to prevent cognitive impairment in patients with type 2 diabetes without obesity.

The potential mechanisms underlying the diabetes-related cognitive impairment include multifactorial pathways implicated in hyperglycemia-induced oxidative stress, neuroinflammation, vascular diseases, central insulin resistance, amyloid-β accumulation, and the consequent development of neurodegeneration (22–24, 28). Our findings based on the HbA_1c_-elevated and -decreased groups also corroborate the possibility that hyperglycemia would be an effective target to reduce the risk of cognitive impairment in type 2 diabetes. In this respect, there are increasingly sophisticated therapeutic approaches for glycemic control in recent years (29), thereby supporting the implementation of strategies to prevent cognitive impairment in type 2 diabetes. Conversely, there are patients with inadequately controlled type 2 diabetes, like those in the HbA_1c_-elevated group in this study, so additional studies are needed to address this issue and develop effective treatment strategies for type 2 diabetes.

**Figure 3:**
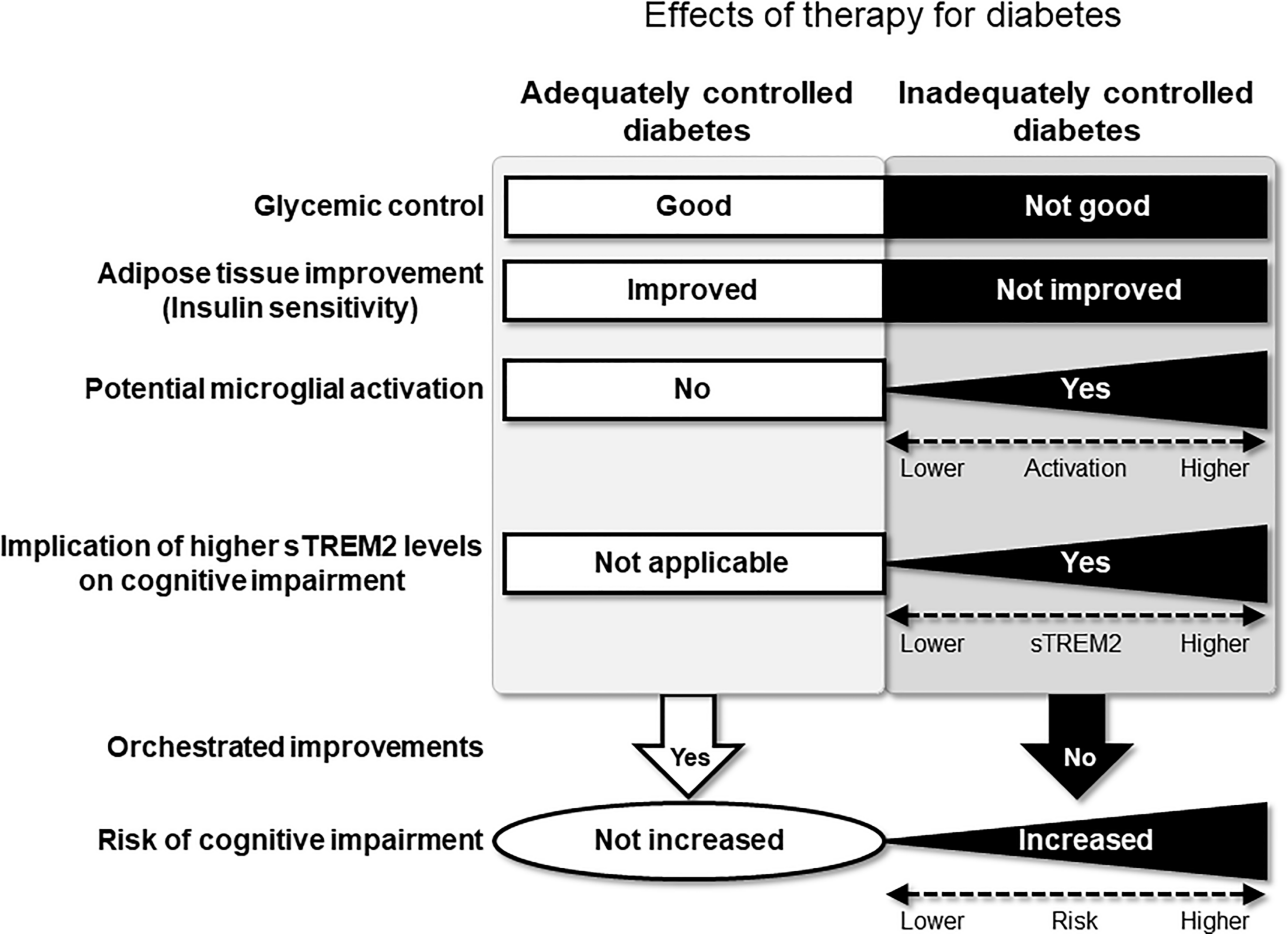
Hypothetical pathological relationships exist between sTREM2 levels, diabetes-related parameters, and cognitive impairment in patients with type 2 diabetes without obesity. Effective therapy for diabetes will allow the orchestrated improvement of risk factors for cognitive impairment. Conversely, in inadequately controlled type 2 diabetes without obesity, sTREM2 derived from the adipose tissue as well as hyperglycemia will activate microglia. The activated microglia can subsequently produce sTREM2, leading to an increased risk of cognitive impairment.

According to our results, it is possible that serum sTREM2 is a novel predictive marker for cognitive impairment in patients with inadequately controlled type 2 diabetes. Although the mechanisms underlying the relationship between sTREM2 and cognitive impairment still remain unclear, our findings suggest potential implications of adipose tissue remodeling in the relationship. Since adipose tissue expresses TREM2 (19), higher levels of sTREM2 would reflect a greater amount of adipose tissue. Thus, a greater reduction of adipose tissue would result in a greater reduction in sTREM2 levels. In this respect, it is possible that the effects of diabetes management on reducing WC and sTREM2 levels were more evident in patients with a greater amount of adipose tissue in the HbA_1c_-decreased group in this study. Accordingly, higher sTREM2 levels at baseline were associated with a greater tendency for reduction in WC and sTREM2 levels at the 2-year follow-up in this group. In conjunction with a reduction in WC, our results further showed that higher sTREM2 levels at baseline were also associated with a greater tendency for reduction in HOMA-R, an indicator of insulin resistance which is a risk factor for cognitive impairment (22–24). Overall, these findings suggest that 2 years of diabetes management were effective in improving adipose tissue, sTREM2 levels, and insulin resistance, thereby exhibiting potential preventive effects on cognitive impairment in the HbA_1c_-decreased group. In contrast, these orchestrated improvements were not observed in the HbA_1c_-elevated group. Therefore, in addition to glycemic control, reducing adipose tissue could contribute to preventing cognitive impairment in type 2 diabetes; sTREM2 and its producing tissue, adipose tissue, could be novel targets for reducing the risk of cognitive impairment in type 2 diabetes (**Figure 3**).

Importantly, it still remains controversial whether sTREM2, as well as TREM2-expressing microglia, exhibits beneficial or detrimental effects on cognitive function (9, 10, 30–33); sTREM2 activates microglia to produce proinflammatory cytokines, but it also reduces amyloid-β deposition (33). However, sTREM2 levels in the blood have been suggested to reflect microglial activation (15). It has also been reported that sTREM2 in the blood would pass through the damaged blood–brain barrier and enter the brain, and *vice versa*, in patients with AD (34), and that there is a positive association between sTREM2 levels in the blood and those in CSF in patients with AD (35). Furthermore, diabetes reportedly reduces the integrity of the blood–brain barrier (22). Therefore, it is possible that sTREM2 produced in adipose tissue might enter the brain from the blood and activate microglia, thereby leading to cognitive impairment in diabetic conditions. In this respect, our findings suggest that the sTREM2-mediated brain–adipose tissue axis might have a novel pathological implication in cognitive impairment in type 2 diabetes.

Regarding the time course of the changes in AD-related markers, it has been hypothesized that CSF sTREM2 levels do not begin to increase before the elevation of CSF Aβ levels, rather they begin increasing prior to the onset of clinical symptoms from cognitively normal stages to preclinical AD (13). Nevertheless, although the pathological significance of serum sTREM2 levels in the continuum of cognitive impairment in type 2 diabetes remains unclear, an *in vitro* study showed that high levels of glucose activated microglia to up-regulate TREM2 expression and induce an inflammatory response (36). Furthermore, another study reported that diabetic conditions impaired microglial function in both mice and humans (18). Hence, as a possibility that serum sTREM2 are derived from adipose tissue (19) and activated microglia (15) exists, we speculate that hyperglycemia and/or insulin resistance first triggers the elevation of sTREM2, even in a cognitively normal stage in diabetic conditions, ultimately leading to microglial dysfunction and cognitive impairment if diabetic conditions are not improved. Therefore, future studies should address these issues by identifying the main cells and tissue that produce sTREM2 during the disease course and elucidating the pathological roles of sTREM2.

This study has some limitations. First, the follow-up period in this study was 2 years. Although sTREM2 levels at baseline showed a significant association with changes in MMSE scores in the HbA_1c_-elevated group and with those in WC, HOMA-R, and sTREM2 levels in the HbA_1c_-decreased group at the 2-year follow-up, significant associations were not manifested at the 1-year follow-up. Further longitudinal prospective studies with longer follow-up periods and larger sample size would be required to support the findings of this study. Second, WC was measured, but the amount of visceral and subcutaneous adipose tissue was not examined in the patients in this study. Gene expression levels of TREM2 and amount of sTREM2 in these tissues were also not investigated. However, the invasive procedure for adipose tissue collection would be difficult in the light of primary care and clinical office-based practice. Another important issue is the potential relationship between serum sTREM2 levels, metabolic markers, and biomarkers involved in neurodegenerative diseases, such as the amyloid-β_42_/amyloid-β_40_ ratios, p-tau181 levels, and levels of the neurofilament light chain. Therefore, future research to elucidate these relationships may lead to novel insights into the pathological significance of sTREM2 in cognitive impairment in type 2 diabetes. In addition, as an index of glycemic control, HbA_1c_ levels at baseline were compared with those at 2-year follow-up in this study. Measurement of more continuous fluctuations of blood glucose would be helpful for corroborating the findings of this study. Finally, the function of sTREM2 in cognitive impairment in type 2 diabetes still remains unclear. Future basic and clinical studies to address these issues would provide a deeper understanding of the significance of targeting sTREM2 and/or adipose tissue for the prevention of cognitive impairment in type 2 diabetes.

In conclusion, the present study provided the first evidence that higher levels of serum sTREM2 would be a potential novel marker for predicting cognitive impairment in patients with inadequately controlled type 2 diabetes without obesity. Accordingly, measuring serum sTREM2 levels would allow the identification of patients with increased risk of cognitive impairment, thereby reducing the risk of cognitive impairment by intensifying diabetes therapy in these patients. Our findings further suggest that sTREM2, as well as hyperglycemia, are effective targets for the prevention of cognitive impairment in type 2 diabetes without obesity. Further experimental and cohort studies to elucidate the pathophysiological significance of sTREM2 will contribute in developing novel effective strategies to prevent cognitive impairment in type 2 diabetes.

## Data Availability Statement

The original contributions presented in the study are included in the article/**Supplementary Material**. Further inquiries can be directed to the corresponding authors.

## Ethics Statement

The studies involving human participants were reviewed and approved by the Central Ethics Committee for Clinical Research at the National Hospital Organization headquarters. The patients/participants provided their written informed consent to participate in this study.

## Author Contributions

MT and HY researched the data, contributed to the discussion, and wrote and reviewed/edited the manuscript. KM, TY, RA, AO, YM, TW, MiS, SK, KY, TT, MaS, MoS, MM, MoN, TK, HW, KH, KK, and MiN researched the data, contributed to the discussion, and reviewed/edited the manuscript. NS-A researched the data, contributed to the discussion, and wrote and reviewed/edited the manuscript. All authors contributed to the article and approved the submitted version.

## Funding

This work was supported in part by Grant-in-Aid for Scientific Research (C) to MT (JSPS KAKENHI Grant Number JP19K07927), HY (JP19K07905), and (B) to NS-A (JP18H02737 and 21H02835), and by Grant-in-Aid for Exploratory Research to NS-A (JP18K19769) from Japan Society for the Promotion of Science. This study was also supported in part by a grant from Takeda Science Foundation to MT; a grant from Health Science University to MT.; a grant from Smoking Research Foundation to NS-A (2019T004); and a grant from the National Hospital Organization for collaborative clinical research to NS-A (H26-NHO-02 and H29-NHO-01). The funders had no role in the design of the study; in the collection, analyses, or interpretation of data; in the writing of the manuscript, or in the decision to publish the results.

## Conflict of Interest

The authors declare that the research was conducted in the absence of any commercial or financial relationships that could be construed as a potential conflict of interest.

## Publisher’s Note

All claims expressed in this article are solely those of the authors and do not necessarily represent those of their affiliated organizations, or those of the publisher, the editors and the reviewers. Any product that may be evaluated in this article, or claim that may be made by its manufacturer, is not guaranteed or endorsed by the publisher.
