# Supplementary material for: Higher Serum Soluble TREM2 as a Potential Indicative Biomarker for Cognitive Impairment in Inadequately Controlled Type 2 Diabetes Without Obesity: The DOR-KyotoJ-1
Source: Front Endocrinol (Lausanne). 2022 May 3;13:880148. doi: 10.3389/fendo.2022.880148 (PMC9110765; doi:10.3389/fendo.2022.880148)
Supplement: Supplementary file 1 [file Table_1.docx]

Supplementary Material

**SUPPLEMENTARY TABLE S1 |** Changes in parameters from baseline to the 1-year and 2-year follow-up.

|  | HbA_1c_-decreased group | HbA_1c_-elevated group | *P*-value |
| --- | --- | --- | --- |
| N | 68 | 98 |  |
| Changes from baseline to 1-year |  |  |  |
| Δ FPG (mmol/L) | −0.0 ± 3.4 | 0.9 ± 2.9 | 0.058 |
| Δ IRI (pmol/L) | 5 ± 70 | 18 ± 84 | 0.312 |
| Δ HOMA-R | 0.1 ± 5.6 | 1.7 ± 6.1 | 0.148 |
| Δ hsCRP (μg/mL) | −0.1 ± 1.9 | 0.3 ± 3.3 | 0.115 |
| Δ sTREM2 (pg/mL) | 85.1 ± 269.2 | 124.5 ± 306.6 | 0.428 |
| Δ MMSE | 0.2 ± 2.1 | −0.1 ± 2.0 | 0.192 |
|  |  |  |  |
| Changes from baseline to 2-year |  |  |  |
| Δ FPG (mmol/L) | −0.6 ± 3.1 | 0.7 ± 2.6 | 0.009 |
| Δ IRI (pmol/L) | 5 ± 89 | 12 ± 94 | 0.467 |
| Δ HOMA-R | 0.1 ± 4.9 | 1.2 ± 5.6 | 0.013 |
| Δ hsCRP (μg/mL) | −0.1 ± 1.8 | 0.1 ± 2.0 | 0.897 |
| Δ sTREM2 (pg/mL) | 229.7 ± 270.0 | 239.5 ± 339.1 | 0.975 |
| Δ MMSE | 0.2 ± 1.9 | −0.1 ± 2.0 | 0.099 |

Data are expressed as mean ± standard deviation. HbA_1c_: hemoglobin A_1c_, FPG: fasting plasma glucose, IRI: immunoreactive insulin, HOMA-R: homeostasis model assessment ratio, hsCRP: high-sensitive C-reactive protein, sTREM2: a soluble form of triggering receptor expressed on myeloid cells 2, MMSE: mini-mental state examination. Δ represents the difference between the 1- or 2-year and baseline values. *P*-values for trend tests were adjusted for age and gender.
